# Graft Dimensions and Clinical Outcomes Following Anterior Cruciate Ligament Reconstruction Using Hamstring Versus Peroneus Longus Autografts in Graft-Commensurate, Short-Statured Patients: A Prospective Observational Study

**DOI:** 10.7759/cureus.107965

**Published:** 2026-04-29

**Authors:** Kanthi Kiran Kumar Koppolu, Lalithmohan Chodavarapu, Sreekanth Kashayi-Chowdojirao, Venkatesham Bitla, Asif Hussain Khazi Syed

**Affiliations:** 1 Department of Orthopaedics, Nizam's Institute of Medical Sciences, Hyderabad, IND

**Keywords:** anterior cruciate ligament (acl), functional outcome of acl reconstruction, graft diameter, hamstring tendon grafts, peroneus longus tendon graft, short stature

## Abstract

Background

Anterior cruciate ligament (ACL) reconstruction with hamstring tendon autografts is well-established, but peroneus longus autografts have emerged as a promising alternative. Optimal graft diameter is a prerequisite for knee stability and satisfactory postoperative outcomes. Attainable autograft diameter is shown to directly correlate with patient height. The relative efficacy of hamstring (HS-R) versus peroneus longus (PL-R) reconstruction for ACL tears among graft-commensurate, short-statured patients is unknown.

Methodology

In this prospective observational study, 44 patients with a height of 173 cm or less who underwent arthroscopic single-bundle anterior cruciate ligament reconstruction using quadrupled HS-R or tripled PL-R for isolated ACL tears were followed for a minimum of 12 months postoperatively. Patient groups were compared in terms of their demographic and anthropometric characteristics, mode and side of injury, preoperative clinical assessment findings, autograft dimensions attained, and postoperative clinical assessment findings. Donor site morbidity and postoperative complications, where present, were recorded.

Results

The study cohort comprised 22 men each, who underwent HS-R and PL-R. The patient groups were comparable in terms of age, height, weight, body mass index, mode and side of injury, and preoperative International Knee Documentation Committee (IKDC), Lysholm, and Modified Cincinnati Score (MCS). PL-R was associated with significantly longer grafts (median (interquartile range (IQR)): 90 (89.5-90) mm vs. 90 (80-90) mm; *P* = 0.024) with larger diameters (median (IQR): 9 (8-10) mm vs. 8 (8-9) mm; *P* = 0.011) and resulted in lower postoperative thigh hypotrophy (median (IQR): 1 (0-1) cm vs. 2 (1-3) cm; *P* = 0.004) when compared with HS-R. Significant improvements in IKDC, Lysholm, and MCS were observed in both groups following surgery, though their postoperative scores were comparable. All patients in the PL-R group had satisfactory ankle function. However, six patients (27.3%) in the HS-R group reported anterior knee pain and hypoesthesia around the proximal leg.

Conclusions

PL-R is a viable alternative to HS-R for ACL reconstruction, with comparable functional outcomes, but yields longer and thicker autografts. It may be a better graft option in patient subsets with low anticipated graft diameters, such as women and short-statured patients. Given its inevitable use in revision surgery and when hamstring autograft yield is insufficient, arthroscopic surgeons may consider adopting this technique more frequently, particularly in selected patients likely to benefit.

## Introduction

Anterior cruciate ligament (ACL) injuries resulting from non-contact pivoting sports or motor vehicle accidents are frequently encountered in orthopedic practice. Disruption of the ACL results in knee instability, functional impairment, and an increased risk of early joint degeneration [1]. Conservative management and ligament repair have been reported for these injuries in the literature, but they may result in persistent instability and high failure rates, respectively [2-3]. Despite significant advances in surgical techniques over time and room for further improvement, arthroscopic single-bundle ACL reconstruction remains the gold standard of care for young, active patients worldwide [4]. The success of this surgery is determined by graft-bone healing, which is influenced not only by the quality of surgical technique and stability of graft fixation but also by the intrinsic properties of the chosen graft itself [5]. Moreover, minimizing donor-site morbidity and surgical failure rates is a prerequisite for satisfactory patient-reported outcomes.

ACL reconstruction comprises a spectrum of procedures that employ aperture, suspensory, post, or hybrid fixation devices, with auto- or allograft options [5]. Among autograft options for ACL reconstruction, bone-tendon grafts were extremely popular until the 1990s, as they offered excellent biomechanical strength and healing, early return to sport, and low revision rates [6]. Subsequently, all-soft-tissue grafts grew in popularity as they eliminated the risk of injuries to the extensor apparatus of the knee and resulted in lower overall donor site morbidity [1]. The primary challenge with all-soft-tissue grafts stems from the need to attain adequate diameter by folding the graft over itself and creating multiple strands while maintaining sufficient length to enable stable fixation [7]. This challenge is further compounded when treating short-statured Asian patients, given the direct correlation that height has with quadrupled hamstring graft diameter and the prevalence of lower median graft length and diameter values among Asians in comparison with Caucasians [8-12].

Despite evidence in the literature indicating the superiority of the recently introduced technique of peroneus longus autograft reconstruction (PL-R) vis-à-vis the established practice of hamstring tendon reconstruction (HS-R) for ACL tears in terms of attainable graft diameter and intrinsic tensile strength, and the comparable functional outcomes and knee stability offered by both, the latter is predominantly used in clinical practice, probably owing to its long-standing track record and familiarity among most arthroscopy surgeons [13-14]. We hypothesized that the greater graft diameter afforded through PL-R would render it a more consistently reliable surgical option with superior overall clinical outcomes when compared to HS-R for short-statured Indian patients with ACL tears. This study aimed to compare PL-R and HS-R for ACL tears in graft-commensurate, short-statured Indian patients in terms of (1) short-term clinical outcomes, including donor-site morbidity and postoperative complications, and (2) graft dimensions.

## Materials and methods

Defining short stature in the context of the study

For this research, *short stature* was intended to represent a height that is indicative of a commensurate suboptimal quadrupled hamstring autograft diameter in the same patient. In their U.S.-based Multicenter Orthopaedic Outcomes Network (MOON) cohort study of 263 patients, Mariscalco et al. [15] reported that a hamstring autograft diameter ≤8 mm was associated with a 7% revision rate, whereas a graft diameter >8 mm was associated with a 0% revision rate. In a Swedish and Norwegian registry-based study of 18,425 patients, Snaebjörnsson et al. [7] reported that hamstring tendon autografts <8 mm in diameter were associated with an increased risk of revision compared with larger-diameter grafts. Likewise, in a subsequent retrospective review of 394 Chinese patients, Tang et al. [11] reported that a hamstring graft diameter ≥8 mm was associated with a significant reduction in graft failure rates. Given that the literature consistently suggests that a graft diameter <8 mm is suboptimal, the cutoff patient height indicative of an approximately 8-mm graft remains to be determined.

Although it is impossible to predict the exact graft size based upon anthropometric details alone, correlation data from prior research and regression analyses have yielded predictive equations correlating patient height (in cm) and autograft diameter (in mm) in the past [8-9]. Thomas et al. [8] derived the equation $diameter = 4.5 + (0.02 \times height)$ from their study on the influence of anthropometric parameters on quadrupled hamstring graft diameter in ACL reconstruction; based on this equation, a graft diameter of 8 mm corresponds to a height of 175 cm. Likewise, Atbasi et al. [9] reported that the diameter of quadrupled hamstring grafts is a function of patient height, following the equation $height = (13.33 \times diameter) + 66.66$; based on this formula, a graft diameter of 8 mm corresponds to a height of 173.3 cm. Since a patient height ≤173 cm appears to correspond to a graft diameter of less than 8 mm, this value was chosen as the cutoff to define graft-commensurate short stature in this study. At the onset of this research, to our knowledge, no predictive equations applicable to Indian patients in this context were available. Hence, this literature was used to define *short stature*.

Study design and patient selection

This study adheres to the STROBE guidelines for observational studies. Following Institutional Ethics Committee approval, a prospective observational study was undertaken. Consecutive graft-commensurate, short-statured Indian patients aged 18 to 45 years who presented with isolated complete ACL tears and underwent arthroscopic primary single-bundle quadrupled HS-R or tripled PL-R between October 2019 and September 2020 were included. Patients with concomitant bony, meniscal, posterior cruciate ligament, or collateral ligament injuries, as well as those with evidence of knee osteoarthritis, foot or ankle deformities, ankle pathology, bony dysplasia, or a prior history of significant trauma, osteoarticular infection, or prior surgery (including primary ACL reconstruction) to either lower limb, were excluded.

All patients were operated on by the same surgical team at a high-volume tertiary care center. The senior author served as the primary surgeon and had over a decade of experience in arthroscopic knee surgery, with proficiency in harvesting both hamstring and peroneus longus grafts. The grafts were secured using cortical suspensory fixation on the femoral side with EndoButton◊ CL Ultra (Smith+Nephew, Andover, MA) fixed-loop devices and aperture fixation on the tibial side with absorbable Biosure HA (Smith+Nephew) screws in all patients. Informed consent was obtained from all study participants.

Preoperative evaluation, graft selection, surgical technique, and postoperative rehabilitation

The diagnosis of an ACL tear was established after obtaining a detailed history, performing a thorough clinical examination, and confirming findings radiologically. Patient demographic data (age, sex, height, and weight), details of the mode of injury, and presenting symptoms were recorded for all patients. The anterior drawer and Lachman tests were performed to detect ACL tears, along with routine specialized tests to assess for associated meniscal or ligamentous injuries, and the results were recorded. Mid-thigh circumference was measured at the midpoint between the inguinal crease and the superior pole of the patella to assess for hypoplasia. This measurement was performed with the patient standing, the knee flexed to 90°, and weight borne on the contralateral limb. Routine anteroposterior and lateral knee radiographs were obtained to rule out bony injuries, while magnetic resonance imaging (MRI) was performed to confirm the presence of an ACL tear, define its characteristics, and identify concomitant injuries. Once the diagnosis was confirmed and ACL reconstruction was deemed necessary, the International Knee Documentation Committee (IKDC) subjective knee form, Lysholm score, and Modified Cincinnati Knee Rating System (MCS) scores were recorded [16-18].

Subjects included in the study were provided with a standardized patient information sheet presenting the pros and cons of HS-R and PL-R in a language they could understand. The information furnished in the sheet was explained during preoperative outpatient counseling. Patients were given the opportunity to clarify their doubts and were offered surgery using an autograft of their choosing. They were free to disclose their preference until the evening prior to surgery, but only after a mandatory minimum of 24 hours following counseling. This interim period was utilized to obtain routine preoperative evaluation results and necessary fitness assessments and clearances. It additionally provided patients time to consider both options and determine what they deemed most appropriate. All patients were reassured that they were free to choose their preferred treatment and that the quality of their surgical and postoperative care would not be influenced by their decision.

**Figure 1:**
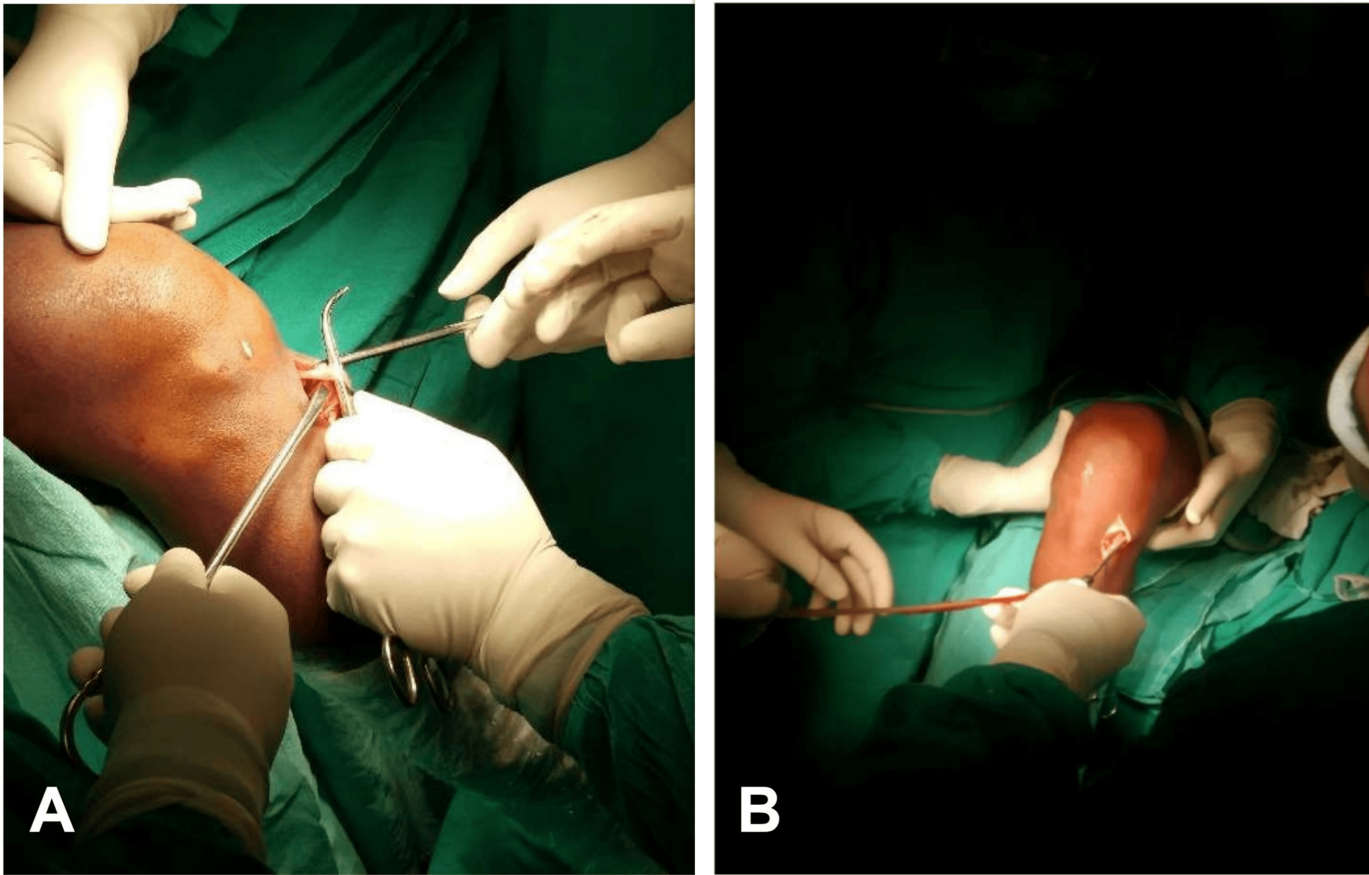
Hamstring tendon harvesting (A) The tendons are identified and freed from surrounding soft-tissue attachments. (B) The graft is shown following harvest and extraction. Source: Authors’ own de-identified intraoperative image.

**Figure 2:**
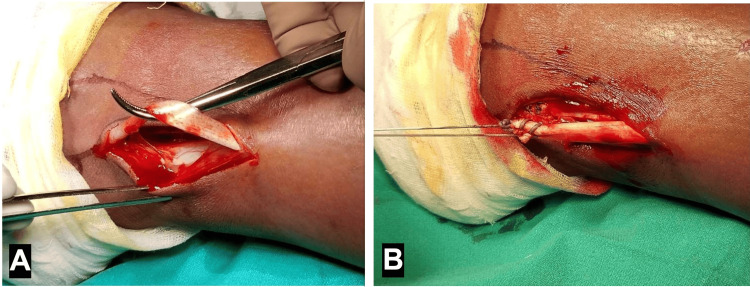
Peroneus longus graft harvesting. (A) The peroneus longus tendon is identified and mobilized. (B) Tenodesis of the peroneus longus to the peroneus brevis is shown. Source: Authors’ own de-identified intraoperative image.

Surgery was routinely performed under spinal anesthesia with a high-thigh tourniquet, with patients in the supine position. A 4-mm, 30º arthroscope and standard anteromedial and anterolateral arthroscopic portals were used. Following diagnostic arthroscopy, the selected autograft was harvested ipsilaterally and prepared. Hamstring graft harvest (Figure 1) began with a 3-4 cm oblique incision over the anteromedial aspect of the proximal tibia at the level of the pes anserinus, followed by incision of the sartorius fascia. Once the gracilis and semitendinosus tendons were identified, they were looped, released from surrounding attachments, harvested using a tendon stripper, debrided, and folded to prepare a four-strand graft. Peroneus longus graft harvest (Figure 2) began with a 1-2 cm incision approximately 1 cm posterior and superior to the lateral malleolus. Once the tendon was identified, it was harvested up to approximately 5 cm distal to the fibular head, debrided, and folded to create a three-strand graft. Tenodesis of the residual peroneus longus to the peroneus brevis was then performed. Graft length and diameter were recorded. Femoral and tibial tunnels were drilled in the anatomic position using standard techniques and cleared of debris, after which the graft was implanted and secured at both ends.

Patients were advised limb elevation, ice fomentation, and ankle pumps starting from the day of surgery. On the first postoperative day, static quadriceps exercises and gentle active knee movement were permitted. Active straight leg raising, hip abduction and adduction, and hamstring strengthening exercises were subsequently encouraged. This was followed by walker-assisted ambulation with weight-bearing as tolerated. The accelerated rehabilitation program described by Shelbourne and Nitz [18] was followed for all study participants. Sutures were removed around the 11th postoperative day. The patients were then followed up at 1, 2, 3, 6, and 12 months postoperatively, and thereafter when necessary. At each follow-up visit, adherence to the rehabilitation protocol was confirmed, and a thorough clinical examination was undertaken. IKDC, MCS, and Lysholm scores, along with the extent of mid-thigh hypotrophy, as well as any signs of donor site morbidity or postoperative complications, were recorded. Additionally, the American Orthopedic Foot and Ankle Society (AOFAS) ankle-hindfoot rating and the Foot and Ankle Disability Index (FADI) were recorded for patients in the PL-R group [19-20]. Appropriate permissions or licenses were obtained for the use of the aforementioned clinical assessment instruments, as required.

Sample size assessment

Given the strict inclusion criteria for this research and the relatively low volume of patients likely to fulfil them, an a priori sample size assessment was imperative. Two challenges, however, needed to be overcome at the time of initiating this study. First, the relative frequencies of suboptimal graft diameters in HS-R versus PL-R for ACL reconstruction among Indian patients were unknown. Second, robust research comparing mean attainable graft diameters in HS-R versus PL-R using a standardized number of graft strands was also unavailable in the literature at that time. However, postoperative IKDC values for each technique were available from the published results of Shi et al. [21]; it was also known from the research by Nwachukwu et al. [22] that the patient-reported minimal clinically important difference (MCID) for the IKDC score following ACL reconstruction is 9 points.

Based on the aforementioned data [21-22], for a study power of 90% with significance set at 0.05, to identify a difference of ≥9 points in postoperative IKDC, a minimum sample size of 5 patients in each limb of the study, totaling 10 patients overall, was adequate. However, a larger sample size was necessary to facilitate better characterization of subgroup data and to adequately distinguish the patient groups in terms of the various parameters assessed. Given the possibility of limited and non-parametric data and the general axiom mandating sample sizes ranging from 5-20 for various non-parametric statistical tests, a minimum of 20 patients in each group was considered more appropriate. Given the possibility of loss to follow-up, 44 patients were therefore recruited overall, with 22 in each group.

Data analysis

Data analysis was carried out using the IBM SPSS Statistics for Windows, version 25.0 (IBM Corp., Armonk, NY) software. Statistical tests were chosen based on the observed data distribution pattern for each variable. Normality of distribution and homogeneity of variance for numerical data were assessed using the Shapiro-Wilk and Levene tests, respectively. Parametric tests were used to analyze normally distributed variables, while non-parametric tests were used when the presumption of normality was violated. Normally distributed numerical variables were expressed as means with standard deviations and compared using the paired or independent samples t-test for related and unrelated datasets, respectively. Numerical variables with non-normal distributions were expressed as medians with interquartile ranges and compared using the Wilcoxon signed-rank test or Mann-Whitney U test for related and unrelated datasets, respectively. Categorical variables were expressed as proportions and compared using Pearson’s chi-square or Fisher’s exact test, as applicable. Spearman’s rank correlation was used to assess the relationship between numerical variables. A *P*-value <0.05 was considered statistically significant.

## Results

All 44 patients included in the study presented with knee instability, had isolated ACL tears confirmed on MRI as well as arthroscopy, underwent surgery using an autograft of their choice, and completed 12 months of postoperative follow-up. Patients in the HS-R and PL-R groups were comparable in terms of demographic characteristics, mode and side of injury, and preoperative clinical assessment findings (Table 1). Although sub-8 mm grafts were encountered in fewer patients in the PL-R group, this difference did not attain statistical significance (Table 2). However, peroneus longus grafts showed not only significantly superior graft length and diameter overall but also less variance in their dimensions when compared with hamstring grafts (Table 2). No significant correlations could be established between graft dimensions and anthropometric characteristics such as height, weight, and body mass index (Table 3).

**Table 1:** A comparison of demographic characteristics and preoperative clinical assessment parameters between patient groups. Numerical data have been expressed as mean ± standard deviation (range) where distribution was normal, and as median (interquartile range) where non-normal distribution was noted. ^*^P-value using the Mann-Whitney U test ^***^P-value pertaining to Fisher’s exact test. ^#^*P*-value pertaining to the independent t-test. ^###^*P*-value pertaining to Pearson’s chi-square test. HS-R, hamstring tendon reconstruction; PL-R, peroneus longus tendon reconstruction; IKDC, International Knee Documentation Committee subjective knee form score; MCS, Modified Cincinnati Knee Rating System score

Parameter	HS-R group	PL-R group	*P*-value
Age (in years)	24.5 (21.75-30.00)	25 (21.75-30.75)	0.906^*^
Sex distribution			
Male, *n* (%)	22 (100%)	22 (100%)	-
Female, *n* (%)	0 (0%)	0 (0%)	-
Height (in cm)	168 (164-170.5)	168 (164-170)	0.868^*^
Weight (in kg)	68 (64-70)	67 (62-76)	0.944^*^
Body mass index	24.1 ± 2.1 (20.8-30.4)	24.1 ± 2.6 (18.8-29.4)	0.985^#^
Side distribution			
Left	13 (59.1%)	11 (50%)	0.545^###^
Right	9 (40.9%)	11 (50%)	-
Mode of injury			
Sport	11 (50%)	11 (50%)	-
Trauma	11 (50%)	11 (50%)	-
Thigh hypotrophy (incidence)			
Present	20 (90.9%)	21 (95.5%)	>0.999^***^
Absent	2 (9.1%)	1 (4.5%)	-
Thigh hypotrophy (in cm)	3 (1.75-4)	2.5 (2-3.25)	0.455^*^
IKDC score	51.5 ± 5.2 (43.7-59.8)	50.4 ± 5.6 (40.2-60.9)	0.511^#^
Lysholm score	49 (45-52)	45.5 (44-54.25)	0.500^*^
MCS	34 (30-34.5)	32 (30-37.5)	0.758^*^
AOFAS	-	95 (92.75-97.5)	-
FADI	-	95 (88-98)	-

**Table 2:** A comparison of graft dimensions between the patient groups. Numerical data have been expressed as mean ± standard deviation (range) where distribution was normal, and as median (interquartile range) where non-normal distribution was noted. *P-value using the Mann-Whitney U test. ***P-value pertaining to Fisher’s exact test. HS-R, hamstring tendon reconstruction; PL-R, peroneus longus tendon reconstruction

Parameter	HS-R group	PL-R group	*P*-value
Graft length (in mm)	90 (80-90)	90 (89.5-90)	0.024^*^
Graft diameter (in mm)	8 (8-9)	9 (8-10)	0.011^*^
Graft diameter (adequacy)			
8 mm or more	19 (86.4%)	21 (95.5%)	0.607^***^
Less than 8 mm	3 (13.6%)	1 (4.5%)	-

**Table 3:** Correlations between graft dimensions and patient demographic parameters. HS-R, hamstring tendon reconstruction; PL-R, peroneus longus tendon reconstruction; Spearman’s r, Spearman’s rank correlation coefficient with its associated *P*-value

Correlation tested	Spearman’s r (HS-R group)	Spearman’s r (PL-R group)
Graft length versus patient height	-0.015 (*P* = 0.949)	-0.113 (*P* = 0.615)
Graft length versus patient weight	-0.051 (*P* = 0.822)	-0.237 (*P* = 0.289)
Graft length versus body mass index	0.079 (*P* = 0.725)	-0.260 (*P* = 0.243)
Graft diameter versus patient height	0.072 (*P* = 0.751)	0.043 (*P* = 0.850)
Graft diameter versus patient weight	0.296 (*P* = 0.181)	0.051 (*P* = 0.823)
Graft diameter versus body mass index	0.153 (*P* = 0.496)	-0.044 (*P* = 0.847)

Postoperative thigh hypotrophy was less prevalent among patients in the PL-R group, although the difference in proportions did not attain statistical significance. Nevertheless, its magnitude was significantly lower in the PL-R group (Table 4). None of the patients in either group had postoperative knee instability or laxity on performing the Lachman test. All patients were able to return to work and perform their daily personal and professional activities within the first year following surgery. All patients were permitted to return to recreational sports at the 1-year follow-up; none were professional athletes. Both study groups were comparable in terms of patient-reported clinical outcome scores (Table 4). Significant improvements in these scores, as well as in thigh muscle recovery, were noted in both groups postoperatively compared with preoperatively (Table 5). All patients in the PL-R group had satisfactory postoperative AOFAS and FADI scores and were free of anterior knee pain. On the other hand, six patients (27.3%) in the HS-R group complained of persistent mild-to-moderate anterior knee pain and hypoesthesia around the proximal aspect of the operated leg. None of the patients developed postoperative infections, knee stiffness, or graft failure.

**Table 4:** Comparison of postoperative clinical outcomes between patient groups. Numerical data have been expressed as mean ± standard deviation (range) where distribution was normal, and as median (interquartile range) where non-normal distribution was noted. ^*^P-value using the Mann-Whitney U test. ^#^*P*-value pertaining to the independent t-test. ^###^*P*-value pertaining to Pearson’s chi-square test. HS-R, hamstring tendon reconstruction; PL-R, peroneus longus tendon reconstruction; IKDC, International Knee Documentation Committee subjective knee form score; MCS, Modified Cincinnati Knee Rating System score; AOFAS, American Orthopedic Foot and Ankle Society ankle-hindfoot rating; FADI, Foot and Ankle Disability Index

Parameters	HS-R group	PL-R group	*P*-value
Thigh hypotrophy (incidence)			
Present	18 (81.8%)	16 (72.7%)	0.472^###^
Absent	4 (18.2%)	6 (27.3%)	-
Thigh hypotrophy (in cm)	2 (1-3)	1 (0-1)	0.004^*^
IKDC score	87.2 ± 9.1 (63.2-97.1)	87.3 ± 8.2 (63.2-95.4)	0.988^#^
Lysholm score	95 (90-100)	95 (92.5-95)	0.617^*^
MCS	91.5 (87.5-95.75)	91 (88.75-96.75)	>0.999^*^
Surgical site morbidity (ankle)			
Postoperative AOFAS	-	94.5 (92.75-97)	-
Postoperative FADI	-	95 (88-98)	-
Surgical site morbidity (knee)			
Anterior knee pain			
Present	6 (27.3%)	0 (0%)	-
Absent	16 (72.7%)	22 (100%)	-
Hypoesthesia around the knee			
Present	6 (27.3%)	0 (0%)	-
Absent	16 (72.7%)	22 (100%)	-

**Table 5:** A summary of postoperative improvements in clinical outcome measures compared with preoperative values among patient groups. HS-R, hamstring tendon reconstruction; PL-R, peroneus longus tendon reconstruction; IKDC, International Knee Documentation Committee subjective knee form score; MCS, Modified Cincinnati Knee Rating System score ^**^P-value using the Wilcoxon signed-rank test. ^##^*P*-value pertaining to the paired t-test.

Parameter	Postoperative improvement (HS-R)	Postoperative improvement (PL-R)
Thigh hypotrophy (in cm)	Significant (*P* = 0.047)^**^	Significant (*P* < 0.001)^ **^
IKDC score	Significant (*P* < 0.001)^##^	Significant (*P* < 0.001)^##^
Lysholm score	Significant (*P* < 0.001)^ **^	Significant (*P* < 0.001)^ **^
MCS	Significant (*P* < 0.001)^ **^	Significant (*P* < 0.001)^ **^
AOFAS	-	Not significant (*P* = 0.102)^ **^
FADI	-	Not significant (*P* = 0.083)^ **^

## Discussion

The principal finding of this study was that PL-R and HS-R resulted in similar postoperative functional outcomes. This was consistent with the results of the meta-analysis by Park et al. [23], as well as Indian studies by Kapoor et al. [24] and Soni et al. [25]. Conversely, a randomized controlled trial by Asif et al. [26] demonstrated significantly better functional outcomes following PL-R compared with HS-R. In another randomized controlled trial by Khalid et al. [27] comparing outcomes of HS-R and PL-R, thigh hypotrophy was found to be significantly less in the latter; our results corroborate this observation. Moreover, in terms of donor site morbidity, PL-R has been shown to be associated with fewer complications than HS-R [23]; our results further validate this inference. Based on the available literature, it can be concluded that ACL reconstruction with peroneus longus grafts is non-inferior, if not superior, to hamstring grafts in terms of postoperative clinical outcomes. To our knowledge, clinical outcomes following ACL reconstruction using peroneus longus and hamstring grafts among short-statured patients have not previously been compared.

The second important finding of this study was that peroneus longus tendons, compared with hamstring tendons, yield autografts with significantly greater length and diameter during ACL reconstruction among graft-commensurate, short-statured Indian patients. The consistency of peroneus longus tendons in this regard was also greater, although statistical significance could not be attained owing to the relatively small sample size for such an assessment. A large meta-analysis by Park et al. [23] demonstrated comparable graft diameters during HS-R and PL-R. Our results, however, are in agreement with several studies comparing outcomes following the use of these graft options in the overall Indian population [24-27]. The reasons for this divergence may be threefold, with patient demography, anthropometric characteristics, and the number of graft strands employed acting as potential confounders and contributing to overall variance. An interesting observation from our results, however, was that 86.4% of patients in the HS-R group attained autograft diameters of 8 mm or more. This might indicate that the mathematical relation between anthropometric measurements and graft size may be population-specific. Further research on different graft options among various demographic groups may shed some light in this regard. Moreover, the statistical significance in median graft diameters between patient subgroups in our study needs to be interpreted because the standard recommendation is to use grafts with a thickness of at least 8 mm, and the consistency of yielding “appropriately sized” grafts was comparable between patient groups in our study. To our knowledge, relative graft dimensions during HS-R and PL-R among short-statured patients have not been studied in the past.

During the course of data collection for the present research, it was noted that an equation $diameter = (0.072 \times height) - 4.003$, correlating patient height (in cm) and doubled peroneus longus tendon autograft diameter (in mm), had been presented in a prospective study by Sakti et al. [28] in the South Sulawesi population. Upon extrapolating this equation to the Indian population, it was observed that a patient height of 166.7 cm was adequate to yield an 8-mm double-stranded peroneus longus autograft. This indicated a strong likelihood of attaining diameters ≥8 mm even among patients considerably shorter than 166.7 cm when the grafts were tripled. This substantial reduction in the patient height is indicative of an acceptably sized graft when considering PL-R as against HS-R, and is corroborated by our findings overall. While these observations are pertinent during ACL reconstruction in short-statured patients in general, they may carry specific implications for women undergoing ACL reconstruction, given that female patients tend to yield hamstring autografts with smaller diameters than their male counterparts [29]. Although our study cohort consisted exclusively of men and precluded any gender-specific conclusions, future research in this regard would be relevant.

Cadaveric and in vivo biomechanical studies have demonstrated comparable to superior tensile strength of the peroneus longus vis-à-vis hamstring tendon autografts [14,30]. Non-inferior, comparable to superior graft dimensions and clinical outcomes have also been reported for the former in the literature [23-27]. However, hamstring autograft reconstruction has remained the preferred modality of treatment for ACL tears for over two decades. The basis for this popularity is likely multifactorial. First, earlier adoption of hamstring grafts resulted in a vast body of literature highlighting their benefits, albeit in comparison with the previous patellar tendon gold standard. Second, for the same reason, several surgeons adopted the procedure earlier and are more familiar and experienced with the technique. Third, subjective experiences may vary, especially during the initial learning curve of a new procedure, leading to surgeon inertia and reluctance to embrace a newer technique.

ACL reconstruction is one of the most common elective procedures in orthopedic surgery. The lack of universal consensus regarding the optimal choice of graft and method of fixation has resulted in a wide range of procedures, with critical surgical decisions often being based on subjective preference and experience rather than objective scientific evidence. Arthroscopic surgeons need to be well-versed in the use of multiple autograft options for ACL reconstruction, especially in settings such as revision surgery and inadequate graft harvest necessitating combined or multiple graft usage. Given the ample room for potential improvement in postoperative outcomes following arthroscopic surgery, and ACL reconstruction in particular, we believe that surgeons should consider using alternative autograft options other than hamstring tendons with increasing frequency, at least in scenarios where the likelihood of patient benefit is high, such as in short-statured or female patients. The importance of a diverse, vast, and robust body of literature supporting these modalities, however, cannot be refuted.

This research is an endeavor in the aforementioned direction, but is not without limitations. The chosen sample size was pre-validated but small out of necessity, which might have been a reason for the failure to demonstrate significant group differences for some of the studied parameters. The duration of follow-up, though adequate, was limited; however, peak outcomes following ACL reconstruction may be expected by this time, since graft healing occurs in a considerably shorter time period, and the need for monitored rehabilitation ceases to exist. A randomized controlled trial design was not opted for since the senior surgeon was motivated by the objective of permitting patients to choose the course of treatment they deemed most appropriate based on their personal perception, requirements, and circumstances. The lack of true randomization may have resulted in the introduction of selection bias. The strengths of this research lie in the novelty of studying short-statured patients and its robust statistical methodology. Future research on this subject, focusing on high-quality evidence, larger sample sizes, and longer follow-up, is essential.

## Conclusions

The peroneus longus is a viable alternative to hamstring tendons for routine ACL reconstruction and offers comparable or superior postoperative outcomes overall in our study. The larger median graft size it provides, with greater consistency as shown in the literature on the Indian population, is a unique advantage, potentially resulting in greater stability and tensile strength. Although it may be a better choice in patient subsets with low anticipated graft diameters, such as women and short-statured patients, further research is needed to clarify this. Given its inevitable use in circumstances such as revision surgery and insufficient hamstring autograft yield, arthroscopic surgeons may benefit from increasing adoption of this technique, at least in selected patients with a high likelihood of benefit.
